
JOURNAL INFORMATION
==============================
NLM Title Abbreviation: Wirtschaftsdienst
Journal ID: Wirtschaftsdienst
NLM Journal ID: 101086612

Wirtschaftsdienst (Hamburg, Germany : 1949)

ISSN: 0043-6275

ARTICLE INFORMATION
==============================
PMCID: PMC7368624
PMID: 32834163
DOI: 10.1007/s10273-020-2689-0
Article ID: 2689
Article version: 1
Subjects: Zeitgespräch

Konjunkturpolitik — post COVID-19: lieber klug statt klotzig

Felbermayr Gabriel J. gabriel.felbermayr@ifw-kiel.de
1
Kooths Stefan stefan.kooths@ifw-kiel.de
2
1 grid.462465.7 0000 0004 0493 2817 Institut für Weltwirtschaft (IfW), Kiellinie 66, 24105 Kiel, Deutschland
2 grid.462465.7 0000 0004 0493 2817 Leiter Prognosezentrum, Institut für Weltwirtschaft (IfW), Kiellinie 66 , 24105 Kiel, Deutschland
Electronic publication date: 2020 Jul 18
Print publication date: 2020
Volume: 100
Issue: 7
First page: 481
Last page: 484
Copyright: © Der/die Autor(en) 2020
License: Open Access Dieser Artikel wird unter der Creative Commons Namensnennung 4.0 International Lizenz veröffentlicht, welche die Nutzung, Vervielfältigung, Bearbeitung, Verbreitung und Wiedergabe in jeglichem Medium und Format erlaubt, sofern Sie den/die ursprünglichen Autor(en) und die Quelle ordnungsgemäß nennen, einen Link zur Creative Commons Lizenz beifügen und angeben, ob Änderungen vorgenommen wurden.
Die in diesem Artikel enthaltenen Bilder und sonstiges Drittmaterial unterliegen ebenfalls der genannten Creative Commons Lizenz, sofern sich aus der Abbildungslegende nichts anderes ergibt. Sofern das betreffende Material nicht unter der genannten Creative Commons Lizenz steht und die betreffende Handlung nicht nach gesetzlichen Vorschriften erlaubt ist, ist für die oben aufgeführten Weiterverwendungen des Materials die Einwilligung des jeweiligen Rechteinhabers einzuholen.
Weitere Details zur Lizenz entnehmen Sie bitte der Lizenzinformation auf http://creativecommons.org/licenses/by/4.0/deed.de.
License URL: https://creativecommons.org/licenses/by/4.0/

issue-copyright-statement: © The Author(s) 2020

==============================

Die Corona-Krise markiert den schärfsten Einbruch der Wirtschaftsleistung seit Bestehen der Bundesrepublik. Niemals zuvor gab die wirtschaftliche Aktivität in Deutschland schneller und drastischer nach als im Frühjahr 2020. War die Industrieproduktion seit Jahresbeginn noch aufwärtsgerichtet, so drückte der Einbruch in der zweiten Märzhälfte das Quartalsergebnis mit 2,5 % deutlich ins Minus. Auch die Dienstleistungsbereiche waren infolge behördlicher Maßnahmen und privater Verhaltensanpassungen schlagartig mit erheblichen Einschränkungen konfrontiert. Insgesamt gab das Bruttoinlandsprodukt (BIP) im ersten Quartal um 2,2 % nach.

Ein Großteil der Shutdown-Maßnahmen blieb den gesamten April 2020 über in Kraft, was insbesondere die auf sozialen Konsum ausgerichteten Dienstleistungsbereiche lähmte. Auch im Exportgeschäft kam es zu drastischen Einbußen. So gaben die Ausfuhren im April fast ein Viertel ihres Vormonatswertes ab. Parallel dazu hat sich die Stimmung in den Unternehmen stark verdüstert: Während die Lageeinschätzung ausweislich des ifo Konjunkturtests binnen weniger Wochen auf das Niveau der Großen Rezession absackte, erreichten die Geschäftserwartungen im April ein Allzeittief. Seitdem zeigen die Stimmungs- und Aktivitätsindikatoren wieder aufwärts, sodass der Tiefpunkt der Krise nun überwunden sein dürfte. Allerdings verläuft die Erholung deutlich langsamer als der vorangegangene Absturz. Im zweiten Quartal dürfte das BIP somit drastisch um 12 % geschrumpft sein. Damit ist in wenigen Monaten die Wirtschaftsleistung doppelt so stark eingebrochen wie während der Großen Rezession im Verlauf von vier Quartalen. Kurzum: Der Einbruch der Wirtschaftsleistung ist in der jüngeren Wirtschaftsgeschichte ohne Beispiel.

## Exporte und Konsum

Die Achillesferse für die Erholung der deutschen Wirtschaft ist das Exportgeschäft, nicht der private Konsum. Das gravierende Ausmaß der Corona-Krise ist zwar nicht zuletzt dem Umstand geschuldet, dass – anders als etwa 2009 – die privaten Konsumausgaben kein stabilisierender, sondern ein treibender Faktor des akuten wirtschaftlichen Einbruchs waren. So rechnen wir am IfW Kiel nach dem Rückgang um 3,2 % im ersten Quartal 2020 – dem bislang größten Minus beim privaten Verbrauch im vereinten Deutschland – mit einem noch weitaus größeren Rückgang für das zweite Quartal von 13 %. Maßgeblich hierfür ist jedoch im Wesentlichen, dass die Konsumenten ihr Einkommen infolge seuchenbezogener Vorsichtsmaßnahmen nicht ausgegeben haben. In der Folge dürfte allein im ersten Halbjahr Kaufkraft in Höhe von fast 80 Mrd. Euro zurückgestaut worden sein, ein Wert, der im Gesamtjahr noch auf 130 Mrd. Euro anschwellen dürfte. Daraus folgt ein sprunghafter Anstieg der privaten Sparquote, die im zweiten Quartal ein Allzeithoch von über 23 % erreicht haben dürfte.

Dieser zuvor noch nie beobachtete Anstieg der Sparquote ist ein klares Indiz für die Blockade von Konsummöglichkeiten. Im Zuge des Wirtschaftseinbruchs dürfte zwar auch das Vorsichtssparen zunehmen, das tatsächliche Ausmaß der Konsumzurückhaltung ist damit aber in keiner Weise erklärbar. Daher wird sich die zurückgestaute Kaufkraft auch in Konsumnachfrage entladen, sobald es die epidemiologischen Umstände erlauben. Dann werden die privaten Konsumausgaben auch ihre stabilisierende Funktion im Konjunkturverlauf wieder entfalten und den Erholungsprozess unterstützen. Die Erholung der privaten Konsumnachfrage ist daher im Wesentlichen eine Frage der Normalisierung der menschlichen Interaktionsmöglichkeiten.

Demgegenüber lastet die globale Wirtschaftskrise zusätzlich und hartnäckiger auf den deutschen Exporten. Auch hier haben zwischenzeitlich seuchenpolitisch bedingte Friktionen den grenzüberschreitenden Handel behindert. Hinzu kommt aber noch, dass die weltweit massiven Produktionsausfälle die Unternehmensbilanzen und in der Folge auch ihre Investitionsbereitschaft noch längere Zeit belasten dürften. Die globale Investitionsschwäche bleibt damit für die vor allem auf Ausrüstungsgüter ausgerichtete deutsche Exportwirtschaft im gesamten Prognosezeitraum ein hemmender Faktor. Dies wird auch das Wiederanziehen der inländischen Unternehmensinvestitionen verzögern. Sowohl die Exporte als auch die heimischen Unternehmensinvestitionen dürften am Ende des Prognosezeitraums noch hinter dem Vorkrisenniveau zurückbleiben, während die privaten Konsumausgaben in der zweiten Hälfte des kommenden Jahres diesen Referenzwert voraussichtlich überschreiten werden.

Der Tiefpunkt der Krise dürfte zwar im April durchschritten worden sein, allerdings waren die Produktionseinbrüche so massiv, dass auch trotz anfänglich recht kräftiger Aufholraten der Erholungsprozess noch über das Jahr 2021 hinaus andauern wird. Zwar wird das Vorkrisenniveau des BIP gemäß IfW-Schätzung gegen Ende des kommenden Jahres wieder erreicht werden. Allerdings dürfte die gesamtwirtschaftliche Aktivität dann immer noch um mehr als 2 % hinter dem Niveau zurückbleiben, das ohne Corona-Krise erreicht worden wäre. Insgesamt taxieren wir den krisenbedingten Ausfall an Wirtschaftsleistung in den Jahren 2020 und 2021 auf über 390 Mrd. Euro. Allein diese Größenordnung macht klar, dass Deutschland – nicht anders als die übrige Welt – deutlich geschwächt aus der Krise hervorgehen wird.

## Wirkungen des Konjunkturpakets

Den finanzpolitischen Impuls des Konjunkturpakets veranschlagen wir auf 2,3 % im Jahr 2020 und 1,4 % im kommenden Jahr. Hierin befinden sich allerdings in erheblichem Umfang Mittel, die bereits in früheren Maßnahmen enthalten bzw. absehbar waren. Dazu zählen die nun als Überbrückungshilfen verlängerten Subventionen für mittlere und kleine Unternehmen sowie die Hilfen des Bundes für die Kommunen. Insgesamt reißt die Corona-Pandemie größere Löcher in die öffentlichen Haushalte als je eine Krise zuvor in der Nachkriegszeit. So beläuft sich das gesamtstaatliche Finanzierungsdefizit 2020 auf 192 Mrd. Euro (5,9 % in Relation zum BIP) und auf 111 Mrd. Euro (3,2 % in Relation zum BIP) im kommenden Jahr. Angesichts des beispiellosen Volumens der finanzpolitischen Reaktion stellt sich umso mehr die Frage, ob die Mittel mit Blick auf den gesamtwirtschaftlichen Stabilisierungseffekt ausreichend zielgerichtet eingesetzt werden. Daran bestehen erhebliche Zweifel. Dies umso mehr, als die Unsicherheit über den weiteren Pandemieverlauf wie auch für die ökonomischen Folgen in den kommenden Quartalen weiterhin sehr hoch ist. Diese Unsicherheit gilt es für die ökonomischen Akteure einzuhegen. Hierzu bedarf es Instrumente, die zielgenau, reaktionsschnell und selbstdosierend sind, um die grundsätzlich marktfähigen Wirtschaftsstrukturen weitgehend unversehrt durch die kommenden Quartale zu bringen.

Die Bundesregierung hat in der Krise frühzeitig reagiert. Mit der Ausweitung der Kurzarbeiterregelung, umfangreichen Liquiditätshilfen und dem Wirtschaftsstabilisierungsfonds wurde zunächst eine Reihe von grundsätzlich geeigneten Instrumenten bereitgestellt. Diese hätten jedoch konsequent auf die Betroffenheit durch die Corona-Krise und weniger auf die Unternehmensgröße abstellen müssen. So fielen mittelgroße Unternehmen (11 bis 249 Mitarbeiter) zunächst durch alle Raster. Die Liquiditätssicherung (Kredit- und Garantieprogramme) war als Akutmaßnahme richtig, allerdings hätte dann in einem zweiten Schritt sichergestellt werden müssen, dass Liquiditätshilfen auch sachgerecht abgerufen werden. Hierzu hätte es ein umfassendes Programm zur Eigenkapitalstärkung bedurft, das es bis heute nicht gibt. Hieraus resultieren eine drohende Insolvenzwelle im Zuge der Krise, eine potenzielle Überschuldung vieler Unternehmen nach der Krise, eine tiefgreifende und möglicherweise bleibende Verunsicherung hinsichtlich Unternehmertums und Selbständigkeit sowie allokationspolitische Verzerrungen aufgrund arbiträrer Ungleichbehandlung von Unternehmen. Diese Punkte belasten die Stabilität des Bankensystems und erschweren ein schnelles Durchstarten nach der Krise, unter Umständen belasten sie sogar nachhaltig das Potenzialwachstum.

Die im Zuge des jüngsten Konjunkturpakets eingeführten Überbrückungshilfen für Unternehmen mit bis zu 249 Beschäftigen adressieren zwar die zuvor existierende Mittelstandslücke und stellen Zuwendungen von bis zu 50.000 Euro zur Deckung von 50 % bis 80 % der Fixkosten zur Verfügung. Der Ansatz, neben Liquiditätshilfen auch Zuwendungen zu gewähren, weist zwar in die richtige Richtung. Zu kritisieren ist aber, dass Unternehmen mit mehr als 249 Beschäftigten außen vor bleiben, die Maßnahme an Umsatzeinbrüchen ansetzt, deren wirtschaftliche Effekte über Branchen und Unternehmen höchst unterschiedlich sein können, keine Anreize gesetzt werden, in der Krise neue Absatzmöglichkeiten zu erschließen, die maximale Höhe der Zuwendung im Verhältnis zur Fixkostenbasis von Unternehmen, insbesondere im fixkostenintensiven Verarbeitenden Gewerbe sehr klein ausfällt, keine Möglichkeit vorgesehen ist, einen angemessenen Unternehmerlohn zu decken und schließlich der Förderzeitraum (Juni bis August) viel zu kurz ist, um der Corona-bedingten Sondersituation gerecht zu werden.

## Alternative Vorschläge des Ifw

Um diese gravierenden Nachteile zu vermeiden, hat das Institut für Weltwirtschaft einen Mechanismus vorgeschlagen, der alle Unternehmen, unabhängig von ihrer Größe und Branchenzugehörigkeit, einheitlich behandelt, die tatsächliche Betroffenheit durch die Krise zum Ausgangspunkt nimmt, Anreize, auch in der Krise Umsätze auszudehnen, verstärkt und sowohl ex ante als auch ex post relativ unbürokratisch einsetzbar wäre (Kooths und Felbermayr, 2020).

Den Ausgangspunkt bilden die im Vorjahr beobachteten Betriebsüberschüsse bzw. Selbständigeneinkommen (BÜSE: Bruttoproduktionswert abzüglich Arbeitnehmerentgelte und Vorleistungen) der Unternehmen, wie sie den Finanzbehörden vorliegen. Diese werden in möglichst eng definierten Branchen (4-Steller) mit den während der Corona-Krise tatsächlich erwirtschafteten BÜSE verglichen und daraus branchenspezifische Einbrüche in % berechnet. Individuellen Unternehmen werden BÜSE im Ausmaß des Branchendurchschnitts anteilig (z. B. 80 %) ersetzt. Bereits erhaltene Leistungen, z. B. aus den Soforthilfen, verrechnen sich automatisch, da sie die Betriebsüberschüsse beeinflussen. Der aktuellen IfW-Konjunkturprognose (Boysen-Hogrefe et al., 2020) zufolge beläuft sich der im laufenden Jahr zu erwartende BÜSE-Einbruch auf brutto rund 140 Mrd. Euro. Hiervon wird etwa die Hälfte durch die verschiedenen Hilfsprogramme ausgeglichen. Würden diese Einbußen zu 80 % ersetzt, entstünden dem Staat Kosten in Höhe von 112 Mrd. Euro.

Hiermit würden jedoch alle übrigen Stabilisierungsmaßnahmen – mit Ausnahme des Kurzarbeitergelds – weitgehend entbehrlich. An ihre Stelle träte ein klar fokussierter Stabilisierungsmechanismus, der dort wirkt, wo die Corona-bedingten Einbrüche am stärksten sind. Demzufolge wird auch der Stabilisierungsbeitrag höher sein als bei weniger zielgenauen Instrumenten. Dies entlastet wiederum den Staat an vielen anderen Stellen. So werden aus den BÜSE auch Kredite bedient. Daher verringern sich durch deren Stabilisierung auch die fiskalischen Lasten aus den Garantie- und Kreditprogrammen der Kreditanstalt für Wiederaufbau oder des Wirtschaftsstabilisierungsfonds; dies reduziert zukünftige Risiken im Bundeshaushalt. Zudem müssten die Zuwendungen versteuert werden, was die Belastung der öffentlichen Kassen weiter reduzierte.

Unternehmen, die schon 2019 keine oder nur geringe BÜSE erwirtschaftet haben, erhielten nur geringe Auszahlungen: dies minimiert Mitnahmeeffekte und verhindert, dass in der Krise Unternehmen gerettet werden, die bereits vor der Krise unrentabel waren; allerdings können Unternehmensneugründungen mit diesem Instrument nicht erfasst werden. Für diesen vergleichsweise kleinen Kreis an Betroffenen wären spezielle Hilfsprogramme aufzusetzen. Die Indizierung auf Branchendurchschnitte und ein Ersatz unterhalb von 100 % bewahren die Anreize der Unternehmen, Anstrengungen zur Krisenbewältigung zu unternehmen, was wiederum makroökonomisch stabilisierend und fiskalisch entlastend wirkt. Der vorgeschlagene Lastenausgleich würde Insolvenzen, die direkt von der Corona-Krise verursacht werden, erheblich minimieren.

Im Vergleich zum systematischen Lastenausgleich gehen steuerliche Verlustvor- und rückträge nicht weit genug. Sie stellen auch keine echten Zuwendungen dar, sondern verbessern vor allem die Liquidität der Unternehmen durch Verschiebungen der Steuerlast. Trotzdem: Schon aus steuersystematischen Gründen sind großzügige Verlustvor- und rückträge jedenfalls geboten; ihre Möglichkeit sollte also deutlich ausgeweitet werden – über das Maß von 5 Mio. bzw. 10 Mio. Euro hinaus, das im jüngsten Konjunkturpaket vorgesehen ist.

## Fokus auf Kernprobleme

Die Krisendiagnose macht deutlich, dass die Stärkung der Masseneinkommen nicht das Kernproblem der deutschen Wirtschaft adressiert und daher auch keinen nennenswerten Stabilisierungsbeitrag liefern kann. Maßnahmen wie das temporäre Absenken der Mehrwertsteuersätze oder Einmalzahlungen (Kinderbonus) sind daher aus stabilisierungspolitischer Sicht wenig wirksam. Insbesondere wird die Mehrwertsteuersenkung zu Vorzieheffekten bei langlebigen Konsumgütern führen. Die Stabilitätspolitik muss aber die Jahre 2020 und 2021 zusammen in den Blick nehmen, da die deutsche Wirtschaft auch im kommenden Jahr noch weit unter ihren Möglichkeiten operiert. Jojo-Effekte in bestimmten Konsumsegmenten sorgen vor diesem Hintergrund eher für Störfeuer als für eine stabile Erholung. Zudem läuft diese Maßnahme – wie alle übrigen Instrumente, die die Unternehmen über den Umweg der Konsumankurbelung stützen sollen – gänzlich ins Leere, sobald der Pandemieverlauf wieder dreht und erneute soziale Distanzierungsmaßnahmen erforderlich werden.

Konjunkturpolitische Eingriffe sollten auch nicht mit industriepolitischen Zielen überfrachtet werden. So wäre den in der Krise besonders betroffenen Gastronomen wenig geholfen, wenn jetzt staatliche Infrastrukturinvestitionen hochgefahren oder Digitalisierungsprogramme subventioniert würden. Damit würden vor allem solche Branchen befeuert, die die Krise am wenigsten spüren. Industrie-, Technologie- und Klimapolitik haben einen ganz anderen Zeithorizont als die Stabilisierungspolitik. Eine erfolgversprechende Politik auf diesen Feldern muss langfristig ausgerichtet sein und vor allem über die Definition eines verlässlichen Rahmens den Unternehmen entsprechende Anpassungsstrategien ermöglichen. Strukturwandel – sofern er überhaupt politisch vorausgesehen werden kann – lässt sich nicht in wenigen Quartalen bewerkstelligen. Die Aufgabe der Konjunkturpolitik ist es, die Wirtschaft so schnell wie möglich zu ihrem langfristigen Produktionspotenzial zurückzuführen, nicht das Produktionspotenzial zu transformieren.

Eindeutig richtig im jüngsten Konjunkturpaket ist die weitergehende Entlastung der Kommunen von durch den Bund beschlossenen Sozialaufgaben. Dies stabilisiert die Kommunalfinanzen und damit einen Großteil der öffentlichen Investitionen, die sonst prozyklisch Krisen verstärken würden.

## Fazit

Alles in allem war die Konjunkturpolitik mit den ersten Entscheidungen auf einem guten Weg, indem sie schnell einer akuten Liquiditätsklemme bei den Unternehmen entgegengewirkt hat. Sie hat sich danach jedoch immer mehr verzettelt. Statt den Liquiditätshilfen konsequent einen allgemeinen Mechanismus zur Eigenkapitalstützung folgen zu lassen, wurden konjunktur- mit strukturpolitischen Maßnahmen vermengt. Zudem hat man mit dem Fokus auf die Masseneinkommen auf das falsche Pferd gesetzt. All diese Instrumente sind blind für die noch schwelenden Risiken, insbesondere einen ungünstigen Pandemieverlauf und eine größere Insolvenzwelle, die auch den Bankensektor in Mitleidenschaft ziehen kann. Statt systematischer Stabilisierung der Unternehmen hat man sich von einer Viel-hilft-viel-Mentalität leiten lassen, die davon ausgeht, dass die Pandemie im Großen und Ganzen ausgestanden ist. Im allgemeinen Ausgabenrausch wurden im Vorfeld des Bundestagswahljahres zudem zentrale Haushaltsgrundsätze missachtet und Kreditermächtigungen beschlossen, ohne zuvor bestehende Reserven aufzuzehren. Auch eine transparente Haushaltspolitik schafft Vertrauen und würde somit ohne fiskalische Kosten einen Stabilitätsbeitrag leisten.

Prof. Gabriel Felbermayr, Ph.D., ist Präsident des Instituts für Weltwirtschaft (IfW) in Kiel und Professor an der Christian-Albrechts-Universität Kiel.

Prof. Dr. Stefan Kooths leitet das Prognosezentrum am IfW in Kiel.


Literatur

Boysen-Hogrefe, J., S. Fiedler, D. Groll, N. Jannsen, S. Kooths und S. Mösle (2020), Deutsche Wirtschaft vor mühsamer Erholung, Kieler Konjunkturberichte, 67 (2020-Q2), Weltkonjunktur im Sommer 2020, Kiel.
Kooths S Felbermayr G Stabilitätspolitik in der Corona-Krise Kiel Policy Brief 2020 138 6
